# Biology-driven therapy advances in high-grade serous ovarian cancer

**DOI:** 10.1172/JCI174013

**Published:** 2024-01-02

**Authors:** Yinu Wang, Alexander James Duval, Mazhar Adli, Daniela Matei

**Affiliations:** 1 Department of Obstetrics and Gynecology and; 2 Driskill Graduate Program, Feinberg School of Medicine, Northwestern University, Chicago, Illinois, USA.; 3 Robert H. Lurie Comprehensive Cancer Center, Chicago, Illinois, USA.; 4 Jesse Brown Veteran Affairs Medical Center, Chicago, Illinois, USA.

## Abstract

Following a period of slow progress, the completion of genome sequencing and the paradigm shift relative to the cell of origin for high grade serous ovarian cancer (HGSOC) led to a new perspective on the biology and therapeutic solutions for this deadly cancer. Experimental models were revisited to address old questions, and improved tools were generated. Additional pathways emerging as drivers of ovarian tumorigenesis and key dependencies for therapeutic targeting, in particular, VEGF-driven angiogenesis and homologous recombination deficiency, were discovered. Molecular profiling of histological subtypes of ovarian cancer defined distinct genetic events for each entity, enabling the first attempts toward personalized treatment. Armed with this knowledge, HGSOC treatment was revised to include new agents. Among them, PARP inhibitors (PARPis) were shown to induce unprecedented improvement in clinical benefit for selected subsets of patients. Research on mechanisms of resistance to PARPis is beginning to discover vulnerabilities and point to new treatment possibilities. This Review highlights these advances, the remaining challenges, and unsolved problems in the field.

## Epidemiology, risk factors, and histological subtypes

Over the past 10 years, we have made progress in understanding the biology of and improving the therapeutic options for ovarian cancer (OC), a disease that has been described by many as a “silent killer” and recognized as the deadliest of gynecologic cancers (1, 2). It is estimated that more than 150,000 OC-related deaths occur annually worldwide (3), while 19,710 new diagnoses and 13,270 deaths are projected in the United States in 2023. For all stages of OC combined, 5-year survival rates range from 36% in non-Hispanic Black women to 47%–48% in non-Hispanic White, Asian-Pacific Islander, and Hispanic women, highlighting existing disparities in outcomes (4).

Several nongenetic risk factors have been associated with OC, the most prominent being age, with half of women with OC being older than 63 years. Menopause, having a late full-term pregnancy or never being pregnant, and lifetime ovulatory years (5) are additional risk factors, while oral contraceptives, multiple pregnancies, and breast feeding have been linked to lower risk, presumably due inhibition of ovulation (6, 7). Less than 20% of all OCs are due to hereditary cancer syndromes caused by mutations in genes involved in DNA damage response, especially *BRCA1*, *BRCA2*, *TP53*, *BRIP1*, *RAD51C*, *RAD51D*, and others (4); therefore, germline assessment of mutation status for key genes is recommended for all new cases.

**Figure 1:**
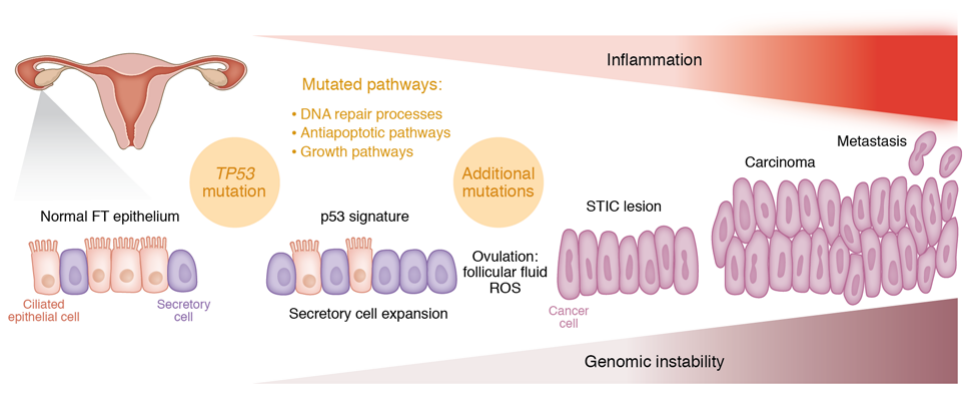
Model of HGSOC initiation from the epithelium of the fallopian tube. Following initiating *TP53* mutation, fallopian tube secretory epithelial cells proliferate and form secretory cell expansion with a *TP53* signature. Follicular fluid released during ovulation contains ROS, which induces inflammation and can cause additional mutations and increased genetic instability. Mutated pathways include DNA repair processes, antiapoptotic pathways, and growth pathways. The secretory cells become irregular in size and shape, and the tissue becomes disordered as serous tubal intraepithelial carcinoma lesions develop. Finally, transformed cells begin to dissociate from the precursor lesion leading to metastasis. FT, fallopian tube; STIC, serous tubal intraepithelial carcinoma; SS-DNA, single-stranded DNA; DS-DNA, double-stranded DNA; MAPK, mitogen-activated protein kinase.

Histologically, ovarian tumors are categorized as epithelial, germ cell, and sex-cord stromal. Epithelial tumors are commonly referred to as OC, and the major histological types are low-grade serous, clear cell, endometrioid, mucinous, and transitional cell (type I) or high-grade serous, mixed epithelial/stromal (carcinosarcoma), and undifferentiated (type II). Unique genetic events characterize each subtype (Figure 1) (8–10). High-grade serous ovarian cancer (HGSOC) accounts for 70% of all OC cases and nearly 80% of deaths.

## Origins of HGSOC

**Figure 2:**
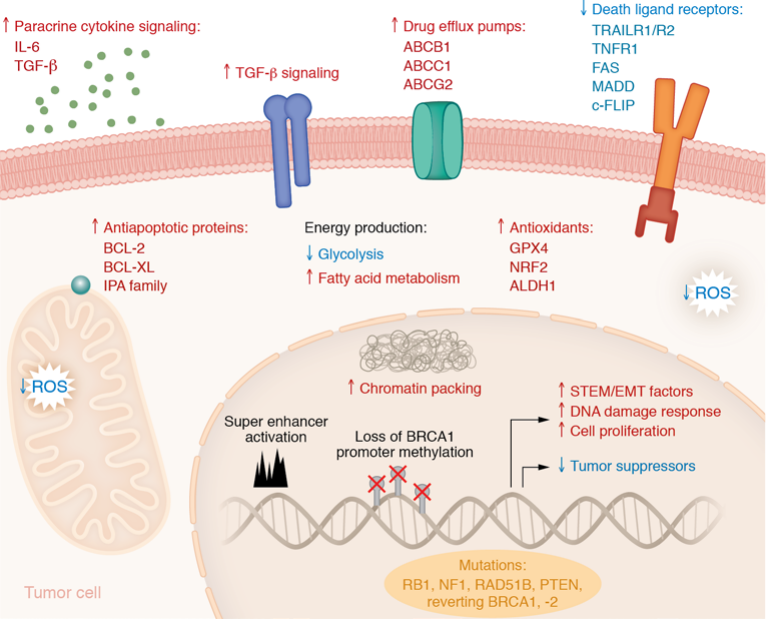
Key mechanisms implicated in emergence of platinum resistance. OC cells develop chemoresistance due to diverse mechanisms, including paracrine release of cytokines from stromal elements in the TME, upregulation of cell membrane ABC transporters to enhance drug efflux, increased cellular antioxidant defense to reduce ROS, promotion of antiapoptotic signaling through increased expression of antiapoptotic proteins and decreased expression of death ligand receptors, metabolic reprogramming, an increase in chromatin packing, genetic and epigenetic inactivation of tumor suppressor and DNA repair genes, modulation of superenhancers that induce transcriptional reprogramming, and acquisition of mutations, including reverting BRCA 1 and 2 mutations. ABCB1, also known as P-glycoprotein (PgP) and multidrug resistance protein 1 (MDR1); ABCC1, multidrug resistance-associated protein 1 (also known as MRP1); ABCG2, breast cancer resistance protein (also known as BCRP); TRAILR1, TNF-related apoptosis-inducing ligand receptor 1; TRAILR2, TNF-related apoptosis-inducing ligand receptor 2; FAS, Fas cell surface death receptor; MADD, MAPK-activating death domain; c-FLIP, cellular FLICE-like inhibitory protein; GPX4, glutathione peroxidase 4; NRF2, nuclear factor erythroid-2 related factor; ALDH1, aldehyde dehydrogenase 1; BRCA1, breast cancer gene 1; EMT, epithelial-mesenchymal transition; RB1, retinoblastoma 1; NF1, neurofibromatosis 1; RAD51B, RAD51 paralog B.

Until the late nineties, OC was thought to originate from the ovarian surface epithelium (OSE), which undergoes repeated cycles of rupture and repair with ovulation. The theory of “incessant ovulation” (11) postulated that OC originates from inclusion cysts, which form as a consequence of repeated rupture of OSE, which allows egg extrusion. Epidemiological studies associating decreased risk of OC with fewer lifetime ovulatory cycles (e.g., due to multiparity, breast feeding, or use of oral contraceptives) (6, 7, 12) supported this concept. The discovery of serous tubal intraepithelial carcinomas (STIC) arising in the distal fimbriae of the fallopian tube and bearing a *TP53*-mutated signature in women carrying *BRCA* mutations (13, 14) caused a paradigm shift, supporting the origin of HGSOC from fallopian tube epithelium (FTE), rather than OSE. Since then, STIC lesions have been recognized as potential HGSOC-precursor lesions that share tumor-specific genetic alterations, such as mutations in *BRCA1*, *BRCA2*, *TP53,* and *PTEN* (15, 16). The concept that HGSOC originates in the secretory cells of the FTE is now at the forefront of the field. Somatic mutation of *TP53* is thought to be the first mutagenic event in the fimbria, is identified in more than 95% of cases of HGSOC, and is shared with STIC lesions. Foci of histologically normal epithelial cells bearing *TP53* mutations are detected in about one-third of healthy tubes of women with *BRCA* mutations, and they are associated with γ-H2AX foci (17), indicating DNA breaks and implicating genomic instability in the early stages of tumor initiation. Emerging studies support the role of oxidative stress in causing genomic instability and promoting tumor initiation in FTE (18). Carcinogenic alterations, such as DNA breaks and TP53 accumulation, and inflammatory changes have been detected ex vivo in cultured FTE cells exposed to follicular fluid or to other oxidants (19, 20). Loss of ciliated cells, which is common with increasing age, is also a risk factor for HGSOC (21), supporting a protective role of these cells in the fimbriae (Figure 2). A recent study using single-cell sequencing of epithelial cells dissociated from the fallopian tube fimbriae identified several expression signatures among the secretory FTE cells, which were recapitulated among the subtypes of HGSOC tumors profiled by The Cancer Genome Atlas (TCGA) (22). Other studies using CRISPR genetic engineering of cancer-related genes such as *TP53*, *BRCA1*, *NF1,* and *PTEN* support the premise that engineered FTE and OSE cells can acquire tumorigenic features, suggesting that HGSOC arises from either the ovary or the fallopian tube (23, 24). However, it should be noted that genetically engineered oviductal cells proliferate and form tumors more aggressively than OSE cells (24). Combined, these data supporting the origin of HGSOC in the fallopian tube fimbriae have led to investigating removal of the tubes (salpingectomy) instead of removal of ovaries (oophorectomy) as preventive surgery for women with high-risk of HGSOC (25). For example, the ongoing SOROCK trial (NCT04251052) compares outcomes of bilateral salpingectomy with delayed oophorectomy to bilateral salpingo-oophorectomy to reduce the risk of OC in women with germline *BRCA* mutations.

## Genetic and nongenetic drivers

OC is driven by gain of function, copy number changes, and loss-of-function mutations in critical tumor suppressor genes (TSGs). Specifically in HGSOC, the hallmark genetic alterations are mutations in *TP53* tumor suppressor and DNA repair genes (26). *TP53* is lost in nearly half of human cancers due to loss-of-function mutations. In HGSOC, point mutations are scattered across the gene, indicating mostly loss-of-function alterations. However, hot spot mutations associated with gain of function occur in *TP53*’s DNA binding domain, such as R175 (~9% of HGSOC), R248 (~6%), and R273 (~6%) (27) (https://www.cbioportal.org/). Interestingly the type of *TP53* mutation and TP53 protein levels were shown to have significant effect on drug response in other gynecologic cancers (e.g., endometrial cancer) (28). In addition to *TP53* mutations, recurrent loss-of-function mutations are also observed in *NF1* (20%), *RB1* (17%), and *PTEN* (29). In addition to loss of TSGs, other hallmark genetic alterations with important therapeutic implications in HGSOC reflect mutations in key DNA repair genes. Among those, germline and somatic *BRCA1/2* mutations occur in roughly 17% and 3% of HGSOCs, respectively (30), while several Fanconi anemia genes (*FANCA*, *FANCI*, *FANCL*, and *FANCC*) and DNA damage response genes involved in homology-directed DNA repair (*PALB2,* *ATM*, *ATR*, *CHEK1*, and *CHEK2*) are recurrently mutated in HGSOC (30). Therefore, over 50% of all HGSOC are homologous recombination deficient (HRD), and this biology has critical therapeutic implications, as discussed below. HGSOC is also driven by genomic copy number changes that lead to a gain of function in several critical genes (26, 29). Among these, the most notable are gene level copy number gain in cyclin E1 (*CCNE1*) (31) and *MYC* genes (32, 33). Notably, amplification of CCNE1, a critical regulator of the G_1_/S transition, is directly associated with poor response to chemotherapy (34) and poor overall patient survival (31, 34). Distinct mutations associate with HGSOC (35) and differentiate this subtype from the other OC subtypes (36–38) (Table 1).

In addition to genetic alterations, OC pathogenesis is governed by a network of transcription factors (TFs). Growing evidence suggests various TFs as critical regulators of gene expression programs in OC subtypes, overall tumorigenesis, and therapy response (39–41). For example, Li et al. identified that expression levels of 17 TFs associate with overall survival (OS) in HGSOC (41). While some TFs are shared across OC subtypes, a set of TFs are expressed in a subtype-specific manner (40). For example, while several HOX family members, hormonal nuclear receptors, and MYC are commonly expressed in several subtypes of OC, aberrant activity of BRCA1/2, FOXM1, and MECOM is relatively more restricted to HGSOC (40). Through integrative analysis of cancer-type-specific gene expression and chromatin state programs, Reddy et al. nominated several master TFs for various types of cancers (39). The analyses identified known regulators of OC, including SOX17 and PAX8 (39), both of which interact and promote angiogenesis in OC (42). TFs also govern chemoresistance, either directly, as in the case of BRCA1/2, or indirectly by reprogramming the transcriptional state that enables cells to tolerate chemotherapy (43). Because cancer cells tend to become dependent on a specific set of TFs that control the dysregulated transcriptional programs (44), targeting these transcriptional dependencies has critical therapeutic implications. Fortunately, advances in medicinal chemistry and targeted-protein depletion strategies such as proteolysis-targeting chimera (45) tools render TFs potentially targetable.

## Experimental models

The completion of the genomic characterization of HGSOC through TCGA and new understanding of the cell of origin have caused a paradigm shift in the use of representative experimental models, with increased focus on platforms that recapitulate the molecular features of HGSOC.

## Cell line models

### Human cell lines.

Cell lines derived from human OC tumors and ascites are useful models to study the disease. They are easy to culture, maintain, and manipulate, and they have been established from different subtypes of OC (see Supplemental Table 1; supplemental material available online with this article; https://doi.org/10.1172/JCI174013DS1). A study that investigated 47 presumed OC cell lines from the Cancer Cell Line Encyclopedia defined the lines that harbor the closest genetic similarity to HGSOC by comparing copy number changes, mutations, and mRNA expression profiles with tumors profiled in TCGA (46, 47). *TP53* was found mutated in 62% of cell lines, while *BRCA1* and *BRCA2* were mutated in 6% and 9% of cell lines, respectively. Two conventionally used cell lines (SKOV3 and A2780) were molecularly dissimilar from HGSOC (47), limiting their current use. *TP53* mutations occur in representative cell lines (KURAMOCHI, OVSAHO, SNU119, COV362, OVCAR4, COV318, JHOS4, TYKNU. OVKATE, CAOV4, OAW28, JHOS2, CAOV3, 59M, ONCODG1, FUOV1, NIH-OVCAR3) (47). *BRCA1/2* mutations are detected in KURAMOCHI, COV362, JHOS2, and PEO1 cells lines, while CCNE1 amplification is present in COV318, ONCODG1, FUOV1, and NIH-OVCAR3 cell lines (47).

### Murine cell lines.

The ID8 cell line was derived from C57BL/6 mouse ovarian surface epithelial cells (MOSECs) transformed by serial passage in vitro (48). These transformed MOSECs form metastatic tumors in the peritoneal cavity of immunocompetent mice (48) and have been used to study antitumor immunity (48). As the ID8 model does not harbor the common *Trp53* mutations, CRISPR/Cas9 gene editing generated cells carrying deletions of *Trp53*, *Brca1,* *Brca2,* and other HGSOC-associated mutations. Various derivative ID8 cells with such alterations were established, including ID8 *Trp53*^−/−^ (49), ID8 *Trp53*^−/−^*Brca1*^−/−^, ID8 *Trp53^–/–^BRCA2^–/–^*, ID8 *Trp53*^−/−^*Pten* ^−/−^, and ID8 *Trp53*^−/−^*Nf1*^−/−^ (50, 51). More recently, a panel of murine FTE cells bearing characteristic mutations and able to form tumors with HGSOC histopathology were described previously (52). These cells phenocopy HRD models though combined loss of *Trp53*, *Brca1*, *Pten*, and *Nf1* and overexpression of *Myc* and *Trp53*^R172H^ and phenocopy homologous recombination proficient (HR-proficient) models through loss of *Trp53* and overexpression of *Ccne1*, *Akt2*, *Trp53* ^R172H^, and *Kras* ^G12V^ or *Brd4* or *Smarca4* (Supplemental Table 2).

## Animal models

### Genetically engineered mouse models.

Given the controversies surrounding OC’s cell of origin, genetically engineered mouse models (GEMMs) have been generated by using mutations of driver genes in either FTE or OSE (Table 2). The Cre/*loxP* system allows tissue-specific gene knockin or knockout either through intrabursal injection of the adenovirus-encoding Cre-recombinase (Ad-Cre), which knocks out *LoxP* site–flanked alleles in situ (53), or via selective expression of Cre-recombinase using tissue-specific gene promoters (54). Table 2 summarizes key models. Among them, GEMMs resembling ovarian serous carcinomas developed by using recombinant Ad-Cre in MOSECs include *Apc*^–/–^*Pten*^–/–^ (53), *Trp53*^–/–^*Rb*^–/–^ (24, 55), *Trp53*^–/–*Brca1*–/–*Myc*–/–^ (56), *Pten*^–/–^*Pik3ca(H1047R)* (57), and *Trp53*^–/–*Brca1*–/–^*Rb*^–/–^ (58). GEMMs for other OC subtypes developed using Ad-Cre include ovarian clear cell carcinoma *Arid1a*^–/–^*Pik3ca(H1047R)* (59), ovarian endometrioid cancer *Arid1a*^–/–*Pten–/–Apc*–/–^ (60), and *Arid1a^–/–^Pten* ^–/–^ (61).

Selective expression of Cre-recombinase in the Müllerian duct epithelium can be driven by tissue-specific promoters, such as *Amhr2* (62), *Pax8* (63), and *Ovgp1* (64). *Amhr2* is expressed in OSE and the stromal cells of the ovary, oviduct, and other portions of the female genital tract (62); *Pax8* is expressed in FTE but also in the endometrial epithelium (65); and *Ovgp1* is only expressed in FTE/mouse oviductal epithelium (64). Although *Trp53* mutations are a hallmark of HGSOC, *Trp53* mutations alone rarely drive OC tumorigenesis (66). However, *Pten* loss in FTE induces serous tumorigenesis (63), and *Pax8*-driven *Pten* loss was sufficient to generate endometrioid and serous borderline tumors (67). Other GEMMs developed using this strategy include *Amhr2* promoter–driven, Cre-mediated *Dicer1* and *Pten* double knockout (68); *Pax8-*driven loss of *Brca1,* *Pten*, and *Trp53* together or of *Brca2, Pten*, and *Trp53* together (63); and *Pax8*-*Cre*–driven *Trp53*^–/–^*Pten*^–/–^*Brca2*^–/–^ or *Trp53*^–/–^*Pten*^–/–^*Brca2*^+/-^ (69). Oviductal serous tumors develop in mice engineered to express the simian virus 40 large T antigen under the control of *Ovgp1* (70). Cho and colleagues developed tamoxifen-regulated *Ovgp1*-*iCreER*^T2^ *Trp53*^–/–^, *Pten*^–/–^, and *Brca1*^–/–^ mice, which resulted in tumors resembling HGSOC (64). *Amhr2-Cre*–driven *Pten^–/–^Kras(G12D)* mice expressing the oncogenic mutant form of *Kras* in OSE cells developed low-grade ovarian serous adenocarcinomas (71). Recently CRISPR/Cas9-mediated gene editing was used to develop a GEMM by intrabursal injection of lentivirus targeting *Trp53*, *Brca1*, *Pten*, *Nf1*, and overexpression of *Myc* and the gain-of-function variant *Trp53(R172H)* (52).

### Syngeneic models.

Syngeneic models are useful to study the tumor microenvironment (TME) and antitumor immunity. The common models use murine cell lines (Table 3). Tumors develop i.p. and are associated with ascites, reproducing the clinical features of the disease.

### Xenograft models.

Xenografts employ immunodeficient mice as hosts. Cancer cells implanted subcutaneously, i.p., or orthotopically (intrabursally) yield variable tumorigenicity (72). Intrabursal implantation gives rise to tumors in the ovary and metastatic spread in the peritoneum, reproducing disease development. Because xenograft models grow relatively fast and display little variation, they are commonly used to evaluate effects of gene manipulation or various therapeutic agents. Patient-derived xenografts developed by implanting OC fragments under the renal capsule or i.p. reproduce the original tumor histology and molecular alterations but grow slower, display higher variability, and are subject to drift during serial propagation (73).

## Metastasis and TME

HGSOC is characterized by a unique pattern of invasion-metastasis. Dislodged from the primary sites in the fallopian tube or ovary where tumors initiate, OC cells float in the peritoneal fluid, attach to the mesothelial layer, and invade into the submesothelial matrix to establish secondary lesions. The most common sites of metastasis occur along the peritoneal cavity, in the fat-rich omentum, and on the surface of bowel or other abdominal organs. Metastasis to distant sites such as lung, skin, bone, brain, and intraabdominal organs is rare and occurs via hematogenous dissemination (74).

The interactions between cancer cells and other components of the peritoneal environment govern this pattern of metastasis. The preference of OC cells to metastasize to the omentum is attributed to energy requirements, which are facilitated by the symbiotic relationship between cancer cells and adipocytes. This direct interaction allows transfer of fatty acids which are used for β-oxidation in OC cells (75). As peritoneal implants develop, OC cells are in direct contact with mesothelial cells. The OC cell–mesothelial cell interaction activates the anti-Müllerian hormone axis, which activates immunosuppressive signals and enables tumor growth (76). HGSOC cells secrete cytokines, microRNAs, and other growth factors that activate fibroblasts in the peritoneal milieu (77). In turn, cancer-associated fibroblasts secrete matrix proteins that sustain tumor cell proliferation and chemokines (IL-6, CLC5, CXCL14, CXCL12) that promote epithelial-mesenchymal transition and OC cell dissemination.

To reproduce events associated with peritoneal metastasis, 3D organotypic models have been developed by using omentum-derived primary human mesothelial cells, fibroblasts, and patient-derived extracellular matrix (78). This model recapitulates the human peritoneal microenvironment and reproduces the molecular mechanisms of metastasis. The model can be expanded to include other cell types from the TME, such as adipocytes, immune cells, or macrophages (79, 80). A four-cell culture model, including cancer cells, mesothelial cells, omental fibroblasts, and adipocytes was developed to study cell interactions during metastasis (81). The unique pattern of HGSOC dissemination reinforces the rationale for i.p. administration of chemotherapy, which has improved clinical outcomes.

Ascites accumulation in the peritoneal cavity is a common symptom of the disease. This fluid contains nonadherent tumor cells or multicellular aggregates; a wide range of nontumor cells, including fibroblasts, adipocytes, mesothelial, endothelial, and inflammatory cells; and acellular components, such as cell-free DNA, cytokines, and chemokines (82). Single-cell transcriptional profiling shows substantial variability in the composition and functions of ascites cells (83). An important driver of ascites accumulation is VEGF (84, 85), and therapies that target it such as bevacizumab effectively inhibit ascites formation and have advanced to clinical practice.

## Immune milieu

HGSOC is one interesting case where most evidence suggests that immune modulators would benefit patients, yet immune-targeting strategies have yielded modest results. Importantly, the majority (~55%) of OC tumors are “immune hot,” meaning that they contain high numbers of tumor-infiltrating T cells (TILs) (86). The 5-year survival rate of patients with TIL-containing tumors is higher compared with those with low number of TILs (74% vs. ~12%) (86). Similarly, a high ratio of cytotoxic CD8^+^ TILs to immune-suppressive Tregs is a favorable marker for patients with OC (87). Recent reports showed that expression of the programmed death (PD) ligand PD-L1 on immune cells in the tumor milieu is associated with increased total numbers of TILs and better survival in HGSOC (88, 89). Additionally, it was reported that OC immunogenicity is regulated by a small subset of CD8^+^ TILs that are primed against high-affinity antigens, representing progenitors of tissue-resident memory cells (90). Humoral tumor B cell–produced IgA responses that sensitize tumor cells to killing by T cells were also described previously (91), and tertiary lymphoid structures, detected in HGSOC tumors, predicted response to immune checkpoint inhibitors (ICIs) (92). These clinical findings strongly indicate the value of the antitumor immune response. However, by and large, testing of immune strategies in OC has been disappointing, with modest 5%–15% response rates to single agents (93) and lack of synergy with combination treatments (94).

Cancer cells develop complex mechanisms to evade immune surveillance. To this end, induction of PD-L1 on cancer cells in response to proinflammatory cytokines such as IFN-γ and TNF-α released from T and NK cells is a crucial immune escape mechanism (95–97). The engagement of PD-L1 on antigen-presenting cells and cancer cells with PD-1 receptor on T cells dampen T cell cytotoxicity, leading to exhaustion (98, 99). Importantly, in addition to its immune inhibitory functions, PD-L1 may also contribute to radio- and chemoresistance (95, 100). Although the mechanism of PD-L1–mediated chemoresistance is poorly understood, recent studies highlight a previously unappreciated role of PD-L1 in DNA repair. Recent findings implicate intracellular PD-L1 as a stabilizer of mRNAs from DNA damage–related genes (101), indicating that it could aid the repair of damaged DNA and, hence, contribute to chemoresistance. Biologics that target PD-L1 and PD-1 interactions have elicited impressive antitumor responses and clinical benefits in many cancer types (100) but not in OC.

It has been postulated that potent immunosuppressive signals dominate the HGSOC TME. Key inducers of the “cold” milieu remain controversial. It was reported that T cell function and IFN-γ secretion are blunted in the OC peritoneal milieu; one of the driving mechanisms involved exposure to malignant ascites, which activates the transcription factor XBP1 and induces the ER-stress response (102). Additionally, XBP1 is activated in dendritic cells, causing functional inhibition (103). Lysophosphatidic acid secreted in OC-associated ascites directly affects dendritic cell function (104), contributing to impaired immune responses. Other T cell inhibitory signals come from myeloid cells, including myeloid-derived suppressor cells (MDSCs) and tumor-associated macrophages, which are detected in tumor tissue, ascites, and peripheral blood of women with OC (105). Ascites fluid stimulates expansion of monocytic-MDSCs through a mechanism dependent on IL-6, IL-10, and STAT3 (106), contributing to immunosuppression. In preclinical models, epigenetic modulators, such as combination of histone deacetylase and DNA methyltransferase inhibitors, enhanced response to anti-PD1 antibodies by depleting MDSCs (107). However, epigenetic priming did not induce robust clinical responses to ICIs in early clinical trials (92). Other strategies targeting MDSCs and tumor-associated macrophages are under clinical investigation.

## Advances in therapy

### Standard treatment.

Women with OC are typically approached with tumor cytoreductive surgery, involving removal of gynecologic organs (total abdominal hysterectomy, bilateral salpingo-oophorectomy), lymph nodes, and omentum followed by platinum (Pt)/taxane-based chemotherapy. Cytoreductive surgery that removes most of the tumor mass, leaving behind microscopic or less than 1 mm tumor implants, is known as “optimal debulking” and affects survival (108, 109). Following surgery, the standard regimen of carboplatin and paclitaxel (CP) has withstood the passage of time (110) with minimal modifications. The clinical effect of Pt on disease control is explained by the DNA repair defects and HRD features of HGSOC (46). Substitution of cisplatin with less toxic carboplatin (111) and other modifications of the regimen have been tested. Dose-dense administration of paclitaxel (weekly vs. every three weeks) demonstrated benefit in some but not all populations and was associated with greater hematological toxicity (112, 113). Rooted in the biology of the disease and taking advantage of its abdominal distribution, i.p. administration of chemotherapy (114) and heated i.p. chemotherapy (115) deliver higher doses of chemotherapy in the peritoneal space, where tumors reside. The i.p. treatment induces improved responses and increased survival compared with intravenous drug delivery. Several ongoing trials are evaluating the impact of heated i.p. chemotherapy after cytoreductive surgery. However, difficulty administering i.p. chemotherapy and controversies surrounding optimal patient selection have remained topics of debate, with current clinical practice trends continuing to favor the standard intravenous administration of the CP regimen every three weeks (116).

### Biology-driven advances in treatment.

Treatment advances over the past decade have stemmed from the successful targeting of two key biological drivers of OC: VEGF-driven tumor angiogenesis and HRD. This success followed decade-long attempts to target other signaling pathways ultimately deemed irrelevant, such as the EGFR, HER-2 neu, HER-3, PDGFR, mTOR, HDAC, and RAF pathways, and others (117).

Development of effective VEGF/VEGFR blockade inhibitors led to the initial testing of the humanized neutralizing antibody bevacizumab in recurrent OC. After noting that bevacizumab had remarkable single-agent activity in recurrent OC (118), particularly in patients with ascites (119), several randomized studies tested its effects in combination with chemotherapy in the upfront and recurrent setting. Bevacizumab plus CP induced improvement in progression-free survival (PFS) in women with newly diagnosed OC (120, 121) and prolonged OS in the high-risk groups, e.g., women who underwent suboptimal surgery and those with stage IV disease (121). Bevacizumab also improved the response rate to chemotherapy and the PFS in women with recurrent Pt-sensitive (122) or Pt-resistant HGSOC (123), leading to its FDA approval and widespread use for both upfront and recurrent OC.

On the other hand, the discovery of synthetic lethality induced by PARP inhibitors (PARPi) in BRCA-mutated cancers (124, 125) and the recognition that approximately half of HGSOC tumors harbor genomic features of HRD (46) led to the fervent investigation of PARPi (126–128), resulting in the approval of three agents (olaparib, rucaparib, and niraparib). Although it has been suggested that all patients with HGSOC benefit from PARPi after response to Pt (126), the highest gain was observed in women with BRCA-mutated or HRD tumors (129, 130). The completion of the SOLO-1 trial, which randomized women with BRCA1/2-mutated HGSOC to olaparib versus placebo for 2 years after completion of standard treatment (131, 132), represents a major step toward cure. Maintenance olaparib reduced the risk of progression by 70% (131), and close to half the women treated on this trial were alive and free of disease recurrence at 7 years (132). The magnitude and the duration of benefit from PARPi in this subset of patients remains unprecedented.

Building upon this success, design of effective combination treatments and exploration of mechanisms of resistance advanced to the forefront. The combination of PARPi and antiangiogenic agents was active in preclinical models and in clinical trials (130). Olaparib and bevacizumab combination was approved as a 2-year maintenance strategy after standard treatment in women with newly diagnosed HRD HGSOC. The additive therapeutic effects of the two drugs were attributed to enhanced HRD induced by bevacizumab, leading to improved responses to PARPi. The observation that PARPi elicits release of cytosolic double-stranded DNA in BRCA-mutated OC cells to induce STING activation and enhance antitumor immunity (133) led to speculation that PARPi could enhance the activity of ICIs. The combination of niraparib and pembrolizumab was found to be moderately active in the nonrandomized phase I/II Topacio trial (134), and results of randomized studies (ATHENA, NCT03522246; DUO-O, NCT03737643; and FIRST, NCT03602859) testing combination PARPi and ICIs in the upfront treatment setting for HGSOC are pending. Other combinations of PARPi with ATR kinase, PIK3/AKT kinase, or RAS/RAF/MEK inhibitors are in early phases of development. These agents in combination may circumvent mechanisms of resistance or accentuate HRD (135).

### PARPi resistance.

Much interest has been devoted to understanding mechanisms of resistance to PARPi. Multiple pathways to resistance exist, including restoration of HR capacity through genetic or epigenetic mechanisms or HR-independent mechanisms affecting efflux mechanisms, PARP trapping, and replication fork stabilization. Initial studies identified reverting somatic *BRCA1* and *BRCA2* mutations as a modality of restoring HR function and a mechanism of resistance to both Pt and PARPi (136, 137). Secondary mutations that restore RAD51C and RAD51D function were also detected in tumors progressing during PARPi and linked to resistance (138). Epigenetic restoration of HR function also occurs through loss of *BRCA1* promoter methylation and associates with Pt and PARPi resistance (139, 140). Another mechanism by which BRCA1-mutated cancer cells regain HR function involves the loss of the protein 53BP1, which mediates the switch between repair of double-stranded DNA breaks from HR to nonhomologous end joining. In BRCA-deficient cells also lacking 53BP, ATM-dependent repair of DNA is activated, HR is restored, and cells become resistant to PARPi (141). CRISPR/Cas9 synthetic lethality screens in BRCA1-mutated cells treated with PARPi identified loss of elements of the Shieldin complex as mediators of PARPi resistance (142). The Shieldin complex, acting downstream of 53BP to promote nonhomologous end joining–dependent double-stranded DNA break repair, sensitizes BRCA-deficient cells to Pt and PARPi (143).

Other mechanisms of resistance that do not depend on restoration of HR function include depletion of the E3 ligase TRIP12, which was shown to limit PARP-1 availability (144), or mutations of the enzyme PARP-1 (145). Both situations limit cancer cell killing by restricting PARPi from trapping PARP-1 on damaged DNA. The efforts to understand mechanisms of resistance are critical to finding new ways to target tumors with innate or acquired PARPi resistance. Based on these findings, ongoing studies are testing emerging drugs or PARPi combinations for tumors predicted to be less responsive to PARP inhibition. For example, HR-deficient OC cells, including cells resistant to PARPi, are highly dependent on polymerase θ. Inhibitors of this enzyme, such as novobiocin, an antibiotic developed in the 1950s, induce synthetic lethality either alone or in combination with PARPi (146).

### Pt resistance.

After initial response to Pt-based therapy (147), most women experience relapse, and tumors become Pt resistant and ultimately fatal (147). In recent years, Pt resistance was recognized as the best predictor of resistance to PARPi, further underscoring the associated clinical adverse outcomes (148). Pt causes intrastrand and interstrand DNA cross-links, which trigger cell death if left unrepaired. Mechanisms of resistance have been studied for decades and include altered membrane transport (149), drug-metabolizing enzymes (149), upregulation of antiapoptotic mechanisms, mechanisms of DNA repair or trans-lesion synthesis, activation of epithelial-mesenchymal transition programs (150), enhanced oxidative defense (151), enrichment in cancer stem cell population (151, 152), or induction of metabolic reprogramming (e.g., a shift from glycolysis to increased fatty acids uptake and oxidation; refs. 153, 154) (Figure 2).

One of the major mechanisms contributing to chemoresistance is upregulation of membrane transporter proteins, such as the adenosine triphosphate-binding cassette (ABC) superfamily transporters (155), which enhance drug efflux (149). Within this family, ABCB1 (also known as P-glycoprotein [PgP] and multidrug resistance protein 1 [MDR1]), ABCC1 (also known as multidrug resistance-associated protein 1 [MRP1]), and ABCG2 (also known as breast cancer resistance protein [BCRP]) are three major isoforms associated with chemoresistance (155). Enhancement of antiapoptotic mechanisms related to either the intrinsic or the extrinsic pathways also contribute to chemoresistance (149). For example, activation of the antiapoptotic proteins BCL-2 and BCL-XL, or of inhibitors of apoptosis proteins, such as the IAP family members (XIAP, survivin), prevent activation of the caspase cascade, promoting cell survival and chemoresistance (149).

Accumulated genomic and epigenomic alterations have been described as key contributors to resistance (156, 157). Whole-genome sequencing of tumor and germline DNA samples from 92 patients with primary refractory and paired sensitive and resistant tumors reported inactivating mutations of TSGs, including *RB1*, *NF1*, *RAD51B*, and *PTEN* in resistant tumors (158). Other genomic changes included reversion mutations of germline *BRCA1* or *BRCA2* mutations, loss of *BRCA1* promoter methylation, and promoter fusion induced overexpression of the drug efflux pump MDR1 (158). *CCNE1* amplification, observed in about 19% HGSOC tumors (158), is mutually exclusive with *BRCA1/2* mutations and common in primary resistant or refractory tumors. A recent proteogenomic analysis of Pt-sensitive and Pt-refractory HGSOC tumors identified chromosome 17 (Chr17) loss of heterozygosity (LOH) as the most robust marker of sensitivity to Pt (159). Chr17 LOH was associated with mutant *TP53* transcriptional signature and responsiveness to Pt, while WT TP53 activity correlated with Pt refractoriness (159). The study proposed a 64-protein panel as a predictive model of Pt resistance (159).

Epigenome alterations can cause transcriptional silencing of TSGs and of genes associated with apoptotic responses to chemotherapy, leading to resistance. Mapping of H3K4me3 (active) and H3K27me3 (repressive) histone marks in primary and recurrent HGSOC identified genes marked by bivalent histone marks in primary tumors (160). This set of genes was enriched in known Polycomb complex target genes from embryonic stem cells and prone to acquiring CpG island methylation in recurrent tumors. It was proposed that acquisition of bivalent chromatin marks contributes to a stem cell–like phenotype that provides tumors with a mechanism for rapid adaptation to Pt (160). Increased CpG island methylation in tumor cells was shown to occur via direct response to hits inflicted by Pt (161) or through signals conveyed from the TME. In response to Pt, fibroblasts secrete cytokines (IL-6, TGF-β) that promote epigenetically mediated cancer cell plasticity and transition to a resistant state (152). In other cancer models, multiscale models combining molecular mapping (Hi-C, scRNA-Seq) with live-cell partial wave spectroscopy showed that cancer cells with high chromatin packing scaling were resistant to Pt (162), supporting the role of the state of chromatin in determining responsiveness to chemotherapy.

From a cell population standpoint, two models have been proposed. One model speculates that Pt eliminates sensitive cells, leaving behind cells tolerant to oxidative stress. Such cells may be cancer stem cells or stem-like cells, both of which typically upregulate aldehyde dehydrogenase (ALDH) and are capable of removing ROS, allowing them to survive chemotherapy (163, 164). This population possesses high ALDH expression, the ability to form spheres, increased expression of stemness-associated TFs, and antioxidant capacity (152, 165). Key antioxidant molecules, including glutathione peroxidase 4 (GPX4), nuclear factor erythroid 2-related factor 2 (NRF2) (166), and ALDH1 (167), are upregulated in cancer stem cells and in resistant cells and tumors (151). Notably, small molecules that block ALDH activity (168, 169) resensitize OC cells to chemotherapy by reducing the antioxidant defense and suppressing Pt-induced senescence and stemness features. Likewise, small-molecule inhibitors targeting GPX4 eliminated Pt-resistant cells via ferroptosis, an iron and lipid peroxidation-dependent form of cell death (151).

The second model assumes that any cell within a tumor can undergo reprogramming to become Pt resistant. This assumption is based on recent genome mapping of H3K27ac, which marks enhancer regions in Pt-sensitive and Pt-resistant cell lines (43). Integrated analysis revealed that distal enhancers, superenhancers, and their gene targets govern transcriptional programs in resistant HGSOC, resulting in the upregulation of key cell signaling pathways (e.g., NF-κB, IL-2/STAT5, TGF-β, and WNT) and downregulation of major metabolic pathways (e.g., oxidative phosphorylation, fatty acid metabolism, TCA cycle). The analysis identified known (e.g., *ZEB2, E2F7, MYC, KLF6*, *ELK3*) and novel (*SOX9, HLX, MYBL1, ZNF430, ZNF502*) superenhancer-regulated master TFs as drivers of Pt resistance. Small-molecule epigenetic inhibitors (e.g., bromodomain inhibitor JQ1) targeted these TFs, reversing the resistant phenotype and supporting the reprogramming concept. Epigenetic interventions using hypomethylating agents to reverse Pt resistance have had moderate success in clinical trials for women with recurrent HGSOC (170, 171).

### Exceptional survivors.

On the flip side, there are rare patients with HGSOC who are not cured but who respond repeatedly to Pt and other lines of chemotherapy and survive longer than 10 years (172). The biological determinants of these exceptional survivors could provide clues to improve outcomes for the reminder of the patients. In a recent study, three key factors associated with survival greater than 10 years were the germline genome, presence of tumor somatic mutations, and antitumor immune response (172). Patients whose tumors exhibited co-occurring alterations in DNA repair pathway genes, such as co-occurrence of *BRCA1* and *BRCA2* mutations, or *RB1* and *BRCA1* or *BRCA2* loss-of-function mutations, lived longer (172). Surprisingly, enhanced proliferation marked by overexpression of the cell proliferation–related genes PCNA and Ki67 was observed in tumors from some long-term survivors, probably because increased proliferation rendered tumor cells more susceptible to chemotherapy and reduced their ability to become quiescent (172). Long-term survivors also harbored a high tumor-mutation burden and a higher quantity of predicted neoantigens compared with short and medium-term survivors (172). An active immune TME was noted in some exceptional survivors, including in rare examples in which patients possessed CCNE1-amplified and HR-proficient tumors, demonstrating the power of the immune system in harnessing the progression of potentially resistant tumors (172).

## Future directions

Important advances during the past decade have honed on defining the cell of origin of HGSOC, identifying genomic vulnerabilities to therapies (PARPi), classifying HGSOC tumors as HR deficient or HR proficient for treatment selection, and identifying subsets of ovarian tumors with unique genomic features for which targeted treatment is still evolving, such as with cyclin E–amplified HGSOC and Arid1A-mutated clear cell ovarian carcinoma. Given the accelerated pace of discovery in the field, we anticipate progress in addressing the remaining unmet needs of women with advanced stage OC. There are several areas of interest that remain unsolved and require solutions to diagnostic and treatment dilemmas in OC. First, early diagnosis and prevention of OC continues to be an unresolved issue that has a profound effect on the deadly course of the disease. The development of highly sensitive methods for detecting cell-free DNA and cancer-specific mutations or patterns of tagmentation in systemic circulation (173) open possibilities for detecting cancer with higher accuracy at an early stage, but testing in prospective studies is lagging. Second, while PARPi have affected the outcomes of women with BRCA-mutated or HRD tumors, there remain limited therapeutic options for women with HR-proficient cancers. This subgroup of patients should be subtyped and approached differently, by identifying and blocking other targets, such as cyclin E or c-Myc. For example, clinical testing of CDK2 inhibitors is underway in patients with cyclin E–amplified tumors (174). Third, despite recent advances in therapy, most women with advanced disease relapse and acquire Pt resistance. Development of strategies for this population is critically needed. Recent advances include antibody drug conjugates (ADCs), such as mirvetuximab soravtansine that targets the folate receptor α to deliver an antitubulin toxin. Mirvetuximab soravtansine induced potent antitumor effects and improved PFS and OS compared with standard chemotherapy in women with Pt-resistant OC (175). Other specific surface proteins in OC considered for development of ADCs include mesothelin (MSLN), tumor-associated calcium signal transducer 2 (TROP-2), sodium-dependent phosphate transport protein 2B (NaPi2b), tissue factor (TF), MUC-16 (CA125), activated leukocyte cell adhesion molecule (CD166), Her-2 neu, and others. Observations that the Her-2 neu–targeting ADC trastuzumab deruxtecan is clinically active not only in high Her-2 neu–expressing breast cancer, but also in patients with breast cancer expressing low levels of the receptor (160), have led to interest in testing it for HGSOC. Finally, because strategies targeting immune checkpoints have failed to make an impact in OC, efforts are underway to identify key inducers of the cold milieu and to design combinatorial therapeutic interventions. With biological discoveries driving therapies, the needle is finally moving for deadly HGSOC.

## Supplementary Material

Supplemental data

## Figures and Tables

**Table 3:**
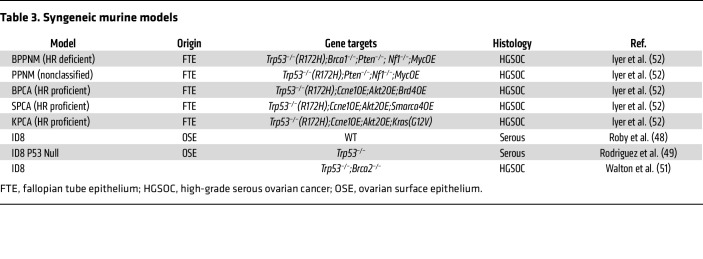
Syngeneic murine models

**Table 2:**
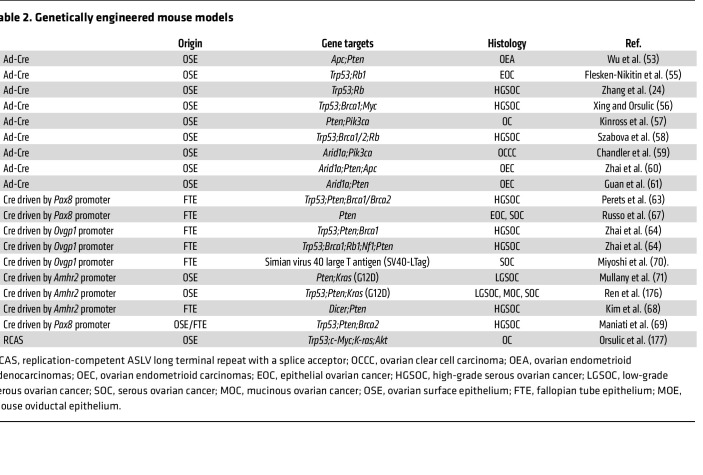
Genetically engineered mouse models

**Table 1:**
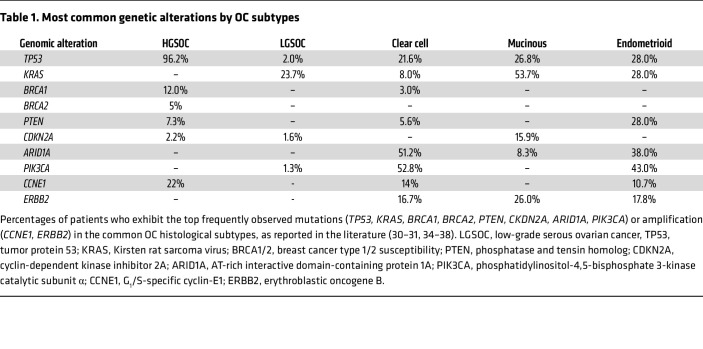
Most common genetic alterations by OC subtypes

## Version 1 01/02/2024

Electronic publication
