# Supplementary material for: Biology-driven therapy advances in high-grade serous ovarian cancer
Source: J Clin Invest. 2024 Jan 2;134(1):e174013. doi: 10.1172/JCI174013 (PMC10760962; doi:10.1172/JCI174013)
Supplement: Supplemental data [file jci-134-174013-s091.pdf]

Supplemental Material:

Supplemental Table S1: Ovarian cancer cell lines

Name	Original annotation	Histology	TP53 status	Other mutations
CAOV3	ovary_adenocarcinoma	HGSOC	Truncating Mutation	
CAOV4	ovary_adenocarcinoma	HGSOC	Missense Mutation	Myc
COV318	ovary_adenocarcinoma	HGSOC	Missense Mutation	CCNE1
COV362	ovary_adenocarcinoma	HGSOC	Missense Mutation	BRCA1, RB1, Myc
FUOV1	ovary_adenocarcinoma	HGSOC	Missense Mutation	Myc, CCNE1
JHOS2	ovary_adenocarcinoma	HGSOC	Truncating Mutation	BRCA1
JHOS4	ovary_adenocarcinoma	HGSOC	Missense Mutation	
KURAMOCHI	ovary_adenocarcinoma	HGSOC	Missense Mutation	BRCA2, Myc, KRAS
NIHOVCAR3	ovary_adenocarcinoma	HGSOC	Missense Mutation	CCNE1, RB1
OAW28	ovary_adenocarcinoma	HGSOC	Truncating Mutation	KRAS
ONCODG1	ovary_adenocarcinoma	HGSOC	Missense Mutation	KRAS, CCNE1, C11orf30
OVCAR4	ovary_adenocarcinoma	HGSOC	Missense Mutation	
OVKATE	ovary_adenocarcinoma	HGSOC	Missense Mutation	
OVSAHO	ovary_adenocarcinoma	HGSOC	Truncating Mutation	BRCA2, RB1
SNU119	ovary_adenocarcinoma	HGSOC	Truncating Mutation	Myc
SNU8	ovary_adenocarcinoma	HGSOC	Truncating Mutation	CCNE1, KRAS
TYKNU	ovary_adenocarcinoma	HGSOC	Missense Mutation	
59M	ovary_adenocarcinoma	HGSOC	Truncating Mutation	Myc
COV504	ovary_adenocarcinoma	HGSOC	Mutation	
OV90	ovary_adenocarcinoma	HGSOC	Missense Mutation	BRAF
OVCAR5	ovary_adenocarcinoma	HGSOC	Wild Type	KRAS
OVCAR8	ovary_adenocarcinoma	HGSOC	Truncating Mutation	ERBB2, KRAS, CTNNB1
OV56	ovary_adenocarcinoma	HGSOC	Truncating Mutation	PTEN,KRAS, ARID1A

RMUGS	ovary_adenocarcinoma	MOC	Missense Mutation	
JHOM1	ovary_adenocarcinoma	MOC	Truncating Mutation	C11orf30, RB1, PIK3CA
COV644	ovary_adenocarcinoma	MOC	Truncating Mutation	
EFO21	ovary_adenocarcinoma	OCCC	Truncating Mutation	
JHOC5	ovary_adenocarcinoma	OCCC	Wild Type	
COLO704	Endometrial_carcinoma	EOC	Wild Type	RB1,PTEN,
OVISE	ovary_adenocarcinoma	OCCC	Wild Type	ARID1A
OAW42	ovary_carcinoma	HGSOC	Wild Type	PTEN,ARID1A
OVTOKO	ovary_adenocarcinoma	OCCC	Wild Type	ARID1A
OVMANA	ovary_adenocarcinoma	OCCC	Wild Type	BRCA2, PIK3CA, ARID1A
RMGI	ovary_adenocarcinoma	OCCC	Homozygous deletion	
HEYA8	ovary_adenocarcinoma	LGSOC	Wild Type	KRAS, BRAF
MCAS	ovary_adenocarcinoma	MOC	Wild Type	PTEN, KRAS
COV434	ovary_adenocarcinoma	EOC	Wild Type	ARID1A
SKOV3	ovary_adenocarcinoma	MOC	Truncating Mutation	PTEN,ERBB2, ARID1A
A2780	ovary_adenocarcinoma	EOC	Wild Type	PTEN, Myc, BRAF, ARID1A
EFO27	ovary_adenocarcinoma	EOC	Truncating Mutation	
OVK18	ovary_adenocarcinoma	EOC	Truncating Mutation	PTEN, KRAS, ARID1A
IGROV1	ovary_adenocarcinoma	EOC & OCCC	Null	PTEN, BRCA1, BRCA2, Myc,ARID1A
JHOC5	ovary_adenocarcinoma	OCCC	Wild Type	BRCA1, BRCA2, Myc, PIK3CA,ARID1A
OC316	ovary_adenocarcinoma	MOC	Null	BRCA2, PIK3CA, ARID1A
OC314	ovary_adenocarcinoma	OCCC	Missense Mutation	Myc, BRAF, ARID1A
TOV21G	ovary_adenocarcinoma	OCCC	Wild Type	PTEN,CTNNB1, PIK3CA, ARID1A
TOV112D	ovary_adenocarcinoma	EOC	Missense Mutation	Myc, PIK3CA, KRAS, CTNNB1, ARID1A
JHOM2B	ovary_adenocarcinoma	MOC	Homozygous deletion/Null	BRCA1, Myc, BRAF
ES2	ovary_adenocarcinoma	OCCC	Truncating Mutation	BRAF,

OV7	ovary_adenocarcinoma	SOC & OCCC	Missense Mutation	KRAS, RB1
NZOV9	ovary_adenocarcinoma	EOC	Wild Type	ARID1A, BRCA2, CTNNB1, PIK3CA, PTEN
OC315	ovary_adenocarcinoma	SOC	Not clear	Not clear
OCIP5X	ovary_adenocarcinoma	SOC	Mutation/ Null	BRAF
SNU840	brenner_tumor	Brenner Tumor	Wild Type	PIK3CA
DOV13	ovary_adenocarcinoma	SOC	Mutation/ Null	
OCIC4P	ovary_adenocarcinoma	OCCC	Mutation/ Null	MSH6, BARD1, CCND1, INPPL1, ERBB3
OCCC: Ovarian clear-cell carcinoma<br>EC: Endometrioid carcinomas<br>HGSOC: High grade serous ovarian cancer<br>LGSOC: Low grade serous ovarian cancer<br>SOC: Serous ovarian cancer<br>MOC:Mucinous ovarian cancer				

Supplemental Table S2. Murine cell lines

Murine established cell line		
Name	Origin	Histology
ID8	MOSE	Serous
IG10	MOSE	Serous
IF5	MOSE	Serous
NuTu-19	MOSE	Serous
BPPNM (HR-deficient)	FTE	HGSOC
PPNM (non-classified)	FTE	HGSOC
BPCA (HR-proficient)	FTE	HGSOC
SPCA (HR-proficient)	FTE	HGSOC
KPCA (HR-proficient)	FTE	HGSOC
